# Assessing the Physico-Mechanical Properties of Three Date Fruit Varieties for Conserving the Keeping and Appearance Qualities

**DOI:** 10.3390/foods14111838

**Published:** 2025-05-22

**Authors:** Mohamed Ghonimy, Raed Alayouni, Garsa Alshehry, Hassan Barakat, Mohamed M. Ibrahim

**Affiliations:** 1 Department of Agricultural and Biosystems Engineering, College of Agriculture and Food, Qassim University, Buraydah 51452, Saudi Arabia; m.elsayed@qu.edu.sa; 2 Department of Food Science and Human Nutrition, College of Agriculture and Food, Qassim University, Buraydah 51452, Saudi Arabia; r.alayouni@qu.edu.sa; 3 Department of Food Science and Nutrition, College of Sciences, Taif University, P.O. Box 11099, Taif 21944, Saudi Arabia; garsa.a@tu.edu.sa; 4 Department of Agricultural Engineering, Faculty of Agriculture, Cairo University, Giza 12613, Egypt; mohamed.ibrahim@agr.cu.edu.eg

**Keywords:** date, friction, fruit, pressure, temperature, physico-mechanical, stress–strain behavior

## Abstract

The physico-mechanical properties of date fruit varieties can indicate their quality and freshness. These properties, which include firmness, moisture content, and mechanical resistance, are closely linked to the fruit’s overall quality and can be used to assess its ripeness and suitability for consumption. Therefore, the current study evaluated the physico-mechanical properties of three date varieties—Sukkari, Khalas, and Saqie—across different ripening stages to enhance food quality and optimize postharvest handling. The study uniquely focused on how ripening stages affect the stress–strain behavior of dates, offering new insights into their mechanical resistance, deformability, and structural stability, all of which are critical parameters for maintaining food quality during storage, transportation, and processing. Significant changes in physical characteristics, including size, mass, moisture content, and density, were observed as the fruit progressed through ripening stages. Sukkari showed the most notable decrease in moisture content, from 61.8% at the Khalal stage to 17.3% at the Tamar stage, resulting in softening and reduced mechanical resistance, potentially impacting shelf life and consumer acceptance. Khalas exhibited a more gradual decline in mechanical properties, with moisture content dropping to 24.6%. At the same time, Saqie demonstrated minimal changes in mechanical properties and moisture content, suggesting better structural and nutritional quality retention. Additionally, the dynamic coefficient of friction increased with temperature and pressure at the Tamr stage, with Sukkari showing the highest values (up to 0.496), followed by Khalas (up to 0.451) and Saqie (up to 0.406). This study introduced the concept of variety-specific differences in frictional behavior, providing valuable insights for improving mechanical processing, reducing physical damage, and preserving date fruits’ nutritional and sensory quality. In conclusion, findings highlight the importance of variety-specific mechanical profiling in improving processing protocols, reducing postharvest losses, and maintaining the nutritional and sensory quality of date fruits for industrial-scale operations.

## 1. Introduction

Date fruits are among the most economically and culturally significant crops in the Arab world and other regions, undergoing substantial physical and mechanical changes throughout the ripening process [1]. Dates are a rich source of nutrients with valuable nutritional benefits and have many uses besides being consumed fresh [2,3]. Date fruits undergo considerable changes in their physical and chemical properties throughout ripening, which affect their processing and quality [4,5]. These include changes in moisture, sugar, and mechanical qualities, including stiffness and flexibility [4,6]. Date mechanical behavior must be understood while building, handling, and processing equipment to reduce damage and maintain quality, as mechanical handling causes microscopic and interior tissue damage to fruits [7]. Date pastes, an intermediate product with sugar substitution potential, are viscoelastic and gel-like, having rheological and tribological differences between cultivars [8]. These properties influence the paste’s texture, lubrication, and potential applications in various food products [8]. Moisture content reductions occur throughout ripening, causing the fruit tissue to become denser and firmer [9]. Stress–strain curves accurately depict how fruit reacts to mechanical forces during handling and processing [10]. Dates progress through distinct ripening stages, each offering unique textures and flavors. The “Khalal” stage marks the initial ripening, when dates are firm, crisp, and brightly colored, often yellow or red, offering a mildly sweet taste. As they mature into the “Rutab” stage, dates become softer, juicier, and sweeter, developing a brownish hue and a tender texture. The final “Tamer” stage is characterized by fully ripened, dried, deep brown, wrinkled, and intensely sweet dates, perfect for long-term storage and consumption [8].

Early Khalal date fruits are more elastic, as stress rises under tension. In the Rutab and Tamr ripening phases, fruits become less elastic and more rigid, resulting in steeper stress–strain curves and a more brittle structure [11]. Traditional mechanical modeling methods like Hertz’s contact mechanics have been used to describe these textural changes and anticipate ripening-related deformation behavior [12]. Changes in the fruit’s physical and mechanical qualities affect the dynamic friction coefficient, which resists sliding [13]. Date fruits like Sukkari, Khalas, and Sagai have smooth, wet surfaces at the Khalal stage, reducing friction and making processing gentle. As the fruit matures through Rutab and Tamr, moisture loss concentrates sugars and fibrous components on the surface, creating rougher, stickier skins that change frictional resistance. These surface changes emphasize the need for ripening stage control during mechanical operations, including pitting, slicing, and packaging [14]. Dynamic friction impacts processing energy consumption and food quality by affecting mechanical damage risk. High friction can produce surface bruising, structural failures, and interior damage, lowering shelf life and marketability [15]. Low friction might create instability during automated processes, misalignment, or incomplete processing. Thus, controlling friction levels among types under diverse environmental circumstances, especially humidity and temperature, is essential for efficient, high-quality processing [16].

Microstructural changes cause frictional variations in fruit skin. Smooth, water-rich surfaces reduce friction in the Khalal stage, helping skin integrity during handling. Dehydration causes microcracking, sugar crystallization, and fiber exposure during ripening, changing the skin’s mechanical contact with processing equipment [17]. Handling tactics should be tailored to the fruit’s microstructure to reduce mechanical stress and preserve food quality during manufacturing. A recent study has shown considerable relationships between the dynamic friction coefficient and mechanical parameters, such as elasticity modulus, toughness, and yield strength [18]. Using Hertzian theory, prediction models may determine fruit toughness, indirectly controlling deformation and friction under operational stresses. Such predictive frameworks can optimize processing line design by assessing dynamic friction, especially in pressure- and temperature-dependent activities like compression molding and thermal treatments [19].

Understanding dynamic friction behavior and equipment design is essential for postharvest handling system improvement. Date fruits are perishable, thus even minimal maltreatment can degrade texture, flavor, and appearance [20]. Using real-time friction monitoring and adaptive control systems during mechanical processing preserves date texture, taste, and customer satisfaction [21]. Date fruit physico-mechanical properties affect keeping and appearance, which are important for consumer satisfaction and industrial processing [22]. The fruit’s durability, texture, and appearance depend on its size, density, moisture content, and mechanical strength. Understanding these traits optimizes storage and processing to preserve the taste and nutrition [6]. Sphericity and roundness affect appearance, with values ranging from 0.63 to 0.69, signifying how round the fruit is [23]. Moisture content is a key factor in the fruit’s texture and shelf life, with values ranging from 10.45% to 18.48% on a dry basis [6]. Mechanical qualities like static friction and crushing strength affect processing. Depending on surface type, the static friction coefficient affects how fruit interacts with processing equipment [22]. Fruit quality and customer preference depend on chemical properties like sugar and minerals. Mechanical and rheological qualities like modulus of elasticity affect customer perception and processing performance by indicating firmness and quality [4].

Previous investigations have established that variations in moisture content, elasticity, and surface texture significantly influence the frictional and mechanical behavior of date fruits across different ripening stages [24]. Since these properties are crucial for maintaining the quality of date fruits, it is also essential to consider the variability among different cultivars. Each variety may require specific handling and storage conditions to optimize its keeping and appearance qualities, highlighting the need for tailored approaches in date fruit processing and marketing. Therefore, assessing both physico-mechanical properties and dynamic friction coefficients is essential for enhancing process efficiency, minimizing losses, and preserving the quality attributes of date fruits throughout the supply chain. The present work aims to evaluate the physico-mechanical properties of date fruits to enhance keeping and appearance qualities.

## 2. Materials and Methods

### 2.1. Fruit Samples and Experimental Design

This study focused on three varieties of date palm fruits—Sukkari, Khalas, and Saqie—at three ripening stages: Khalal, Rutab, and Tamar (Figure S1). The dates were categorized into three stages: Khalal, where fruits are yellow or light brown and still firm; Rutab, marked by soft texture and darkening color; and Tamar, characterized by thoroughly dried fruits with concentrated sugars [17]. A total of 100 fruits per variety and ripening stage were collected manually from a private farm owned by Sheikh Mohammed Abdul Aziz Al-Rajhi, located in Unaizah Governorate, Qassim Province, Saudi Arabia. The geographical coordinates of the farm are approximately 26.0757° N latitude and 43.9916° E longitude, with an elevation of about 650 m above sea level. The region is characterized by an arid climate, with mean annual precipitation of approximately 100 mm, concentrated between November and March. Summer temperatures frequently exceed 45 °C, while winter conditions remain relatively mild. The farm’s soil type is sandy loam, with good drainage and moderate fertility, making it well-suited for date palm cultivation.

Irrigation is carried out through a localized drip irrigation system, using groundwater extracted from on-site wells. To ensure representativeness, fruits were sampled from different parts of the farm to reflect a randomized distribution. On the day of harvesting, the samples were air-shipped to the laboratory within 12 h under carefully controlled conditions. The fruits were packed in ventilated, food-grade plastic containers, cushioned with soft, biodegradable padding to prevent bruising during transit. The containers were then placed inside insulated thermal shipping boxes. During transportation, the internal temperature was maintained at 12–14 °C, with relative humidity levels of 85–90% [25], which are suitable conditions for preserving the physical integrity and quality of date fruits at advanced ripening stages. These conditions were verified using data loggers placed inside the shipping boxes. This approach ensured the samples arrived in optimal condition for subsequent physical and mechanical analysis.

### 2.2. Physical Properties of Date Fruits

**Figure 1:**
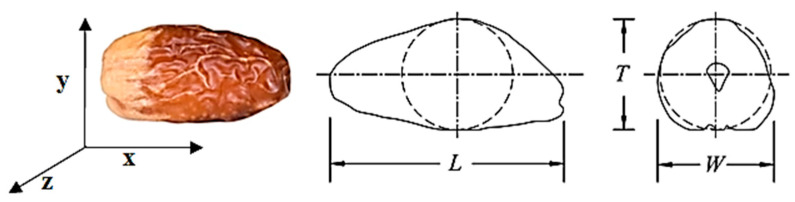
Date fruit dimensions (length, width, and thickness) [18].

Date fruit dimensions (length, L, width, W, and thickness, T) were measured with a Vernier caliper to an accuracy of 0.05 mm. Figure 1 shows the three dimensions of date fruit. The date flesh’s moisture content (MC) was determined using AOAC [26]. The date fruit mass was measured using a digital balance that was accurate to 0.01 g. The fruit bulk and true densities were calculated according to Mohsenin [13]. The fruit geometric mean diameter (d_g_) was calculated from Equation (1) [13].

(1) dg=L·w·T13

Also, the sphericity (S) expresses the shape character of the fruit relative to that of a sphere of the same volume. Assuming that the diameter of the circumscribed sphere is equal to the longest intercept L of the ellipsoid and that the volume of the fruit is equal to the volume of a triaxial ellipsoid with intercepts L, w, and T. Also, the degree of sphericity (S) was calculated from Equation (2) [13].

(2) $$S=\frac{d_{g}}{L}$$

### 2.3. Compression Test and Stress–Strain Curve Analysis

**Figure 2:**
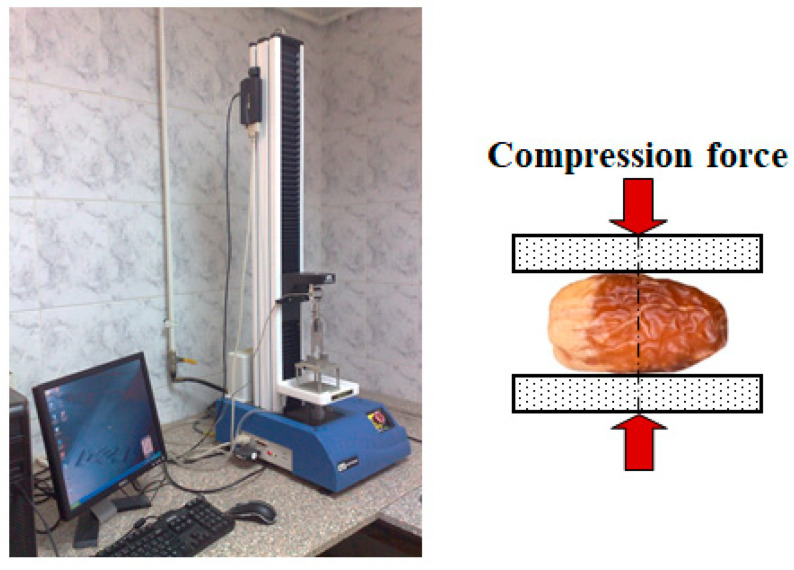
The universal testing machine loaded the date fruit between the two parallel plates.

Uniaxial compression tests were performed on ten randomly selected fruits per variety and ripening stage using an Instron universal testing machine (1000 N capacity, Instron^®^, Norwood, Massachusetts, USA) equipped with parallel stainless plates (75 mm diameter, Figure 2). Prior to testing, fruit samples were conditioned for 24 h at 23 ± 1 °C and 50 ± 5% relative humidity to equilibrate moisture. Fruits were placed longitudinally between the plates to ensure uniform loading along their natural axis, allowing evaluation of their surface and structural response under compression. The initial height of each fruit was measured to an accuracy of 0.1 mm using a digital caliper. This conditioning protocol was selected to simulate typical ambient handling conditions, ensuring that the fruits underwent moisture equilibration similar to that experienced postharvest handling and storage. The chosen relative humidity and temperature conditions were designed to prevent excessive surface dehydration while preserving the internal moisture content, which is crucial for accurately evaluating the fruit’s mechanical properties. These conditions also helped maintain the structural integrity of the fruit, minimizing any potential impact of moisture loss on the results of the uniaxial compression tests. The protocol ensured that the fruit samples maintained a state that closely mirrors real-world conditions, allowing for more reliable and representative testing.

In this study, the manufacturing criteria referred to maintaining the physical and physiological integrity of the date palm fruits during pre-treatment and experimental procedures. Specifically, fruits were selected to be uniform in size, free from mechanical damage, and at the same maturity stage. All handling during pre-treatment (e.g., washing, drying, and coating, if applicable) was performed manually using clean, food-grade tools to avoid bruising or skin rupture. Additionally, temperature and relative humidity were controlled throughout the handling and storage processes, and any fruits showing signs of deterioration or damage were excluded. These steps ensured the experimental results’ reliability and minimized the influence of postharvest stress’s influence.

Compression was applied at a constant crosshead speed of 0.5 mm·s^−1^ until a maximum deflection of 10 mm was reached, while force and displacement data were sampled at 20 Hz using Bluehill^®^ Universal software (version 4.35; Instron^®^, Norwood, MA, USA). To obtain true stress–strain curves, the instantaneous contact area A between the sample and platen was determined at each 0.1 mm increment by lightly inking the upper face of the slab, pressing it onto paper covering the lower plate, scanning the imprint at 600 dpi, and measuring the area with image analysis software.

Instantaneous compressive stress σ and engineering strain ε were calculated point-by-point from Equations (3) and (4).

(3) $$\sigma =\frac{F}{A}$$

(4) $$\varepsilon =\frac{\Delta l}{l_{0}}$$

where F is the recorded force (N), ΔL is the change in sample height (mm), and l_0_ is the original height [13]. For large deformations (ε > 0.1), true strain $\varepsilon_{true}=\ln\left(1+\epsilon \right)$ was also computed. Plotting σ versus ε yielded stress–strain curves for each replicate.

From these curves, elastic modulus E was obtained as the slope of the initial linear region (*dσ/dε*), reflecting tissue stiffness; the yield stress was identified at the end of the linear elastic limit; and the fracture stress and corresponding fracture strain were taken at the peak load immediately before failure. Testing procedures conformed to ASTM D695-15 for compression of rigid materials [27], providing a rigorous framework for deformation-controlled loading of biological tissues. The resulting stress–strain parameters allow direct, geometry-independent comparison of stiffness, strength, and deformability across date varieties and ripening stages, informing the design and optimization of harvesting and postharvest handling equipment.

### 2.4. Dynamic Coefficient of Friction

#### 2.4.1. Device Description

**Figure 3:**
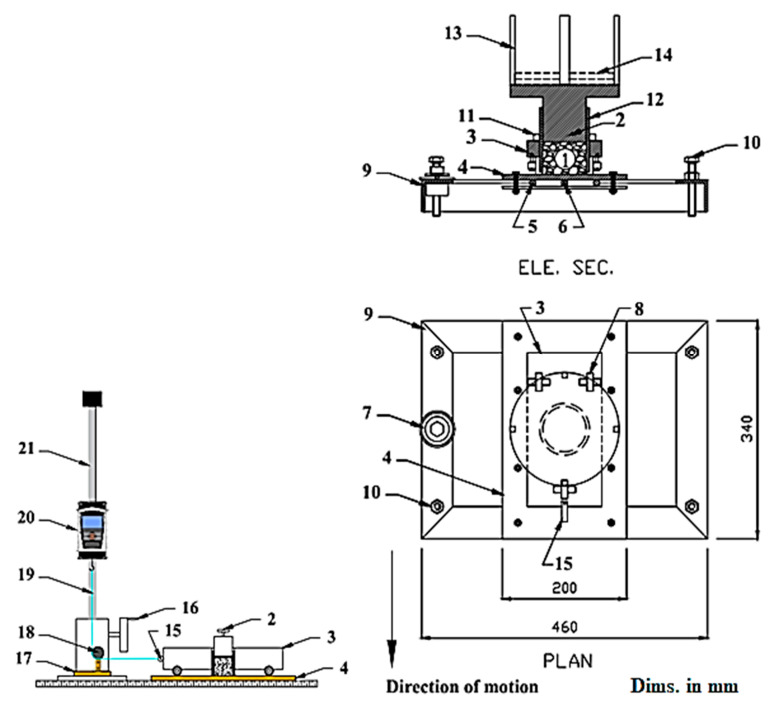

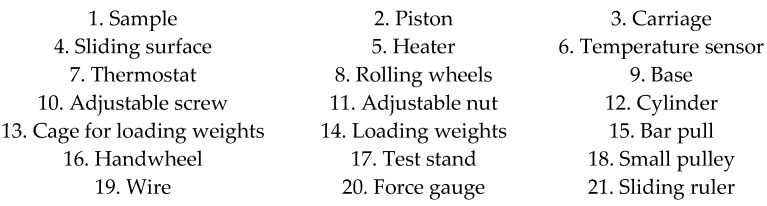
Device for measuring dynamic friction force under varying pressure and temperature.

To facilitate the measurement of the dynamic coefficient of friction (μd) of date fruits under varying pressure and temperature conditions, a specialized mechanical device was developed and assembled for this study. As illustrated in Figure 3, the main structure of the device comprises a vertically adjustable cylindrical chamber with external threading, which allows for precise elevation adjustments via a movable nut. This chamber is mounted on a horizontally sliding carriage supported by three roller-bearing wheels—two at the rear and one at the front—designed to ensure smooth and stable linear movement during testing. Beneath the cylindrical chamber lies a metallic sliding surface made of stainless steel, measuring 200 mm in width, 350 mm in length, and 10 mm in thickness. This surface is mounted on a fixed stainless-steel base with a height of 50 mm, creating space for integrating a heating element and a thermostat underneath, enabling accurate temperature control during experiments.

The cylindrical chamber is partially filled with date fruits and fitted with a cylindrical piston measuring 40 mm in diameter and 70 mm in height. This piston is connected to a steel cage that holds loading weights, which apply a vertical compressive force to the fruit samples, simulating different pressure levels. The entire chamber and loading system rests on the sliding carriage, which is connected to a steel rod attached to a digital force gauge (MARK-10, Model M5-200, capacity 1000 N, Marks Amityville, NY, USA). This gauge records the horizontal force required to initiate movement and maintain steady motion, representing the dynamic frictional resistance.

A specific load is applied to the piston during each test to generate the desired normal pressure. The carriage is then pulled horizontally, and the force required to maintain motion is measured. The difference between the loaded and unloaded force readings is used to compute the dynamic coefficient of friction. This apparatus enables precise control over testing variables and provides reliable data on how temperature and pressure affect the surface interaction properties of date fruits, which is vital for improving postharvest handling and mechanical processing efficiency.

#### 2.4.2. Operating Conditions (Treatments)

The dynamic friction coefficient of three different date fruit varieties was determined specifically at the Tamr ripening stage. This stage was deliberately selected because it is the primary phase during which dates undergo a wide range of postharvest, industrial, and processing operations that often involve applying pressure and heat. Therefore, understanding the frictional behavior of date fruits at the Tamr stage is essential for the proper design, optimization, and efficiency of handling, storage, and processing equipment.

The experiments were carried out under ten different pressure levels (9, 18, 27, 42, 53, 65, 75, 88, 97, and 109 kPa) and six temperature levels (40, 50, 60, 70, 80, and 90 °C), following the methodology outlined by Ranasinghe et al. [28]. Temperature control during the tests was achieved using a built-in thermostat, with temperatures monitored and confirmed through a digital infrared thermometer to ensure accuracy throughout the experiments. These conditions were selected to simulate practical processing environments and assess the influence of thermal and mechanical stresses on the surface behavior of the fruits.

#### 2.4.3. Friction Coefficient Calculation

The dynamic coefficient of friction (*μd*) was computed using Equation (5) [29].

(5) $$\mu d=\frac{F_{f}}{N_{l}}=\frac{F_{T}-F_{E}}{N_{l}}$$

where *μd* is the dynamic coefficient of friction, *F_f_* is the friction force (*Ff* = *F_T_* − *F_E_*), N; *F_T_* is the force required to start motion of the filled wooden frame with the samples, N; *FE* is the force required to start motion of the empty wooden frame, N; and *N_l_* is the normal load (weight) pressing the sample to the contact surface, N. Three replicates were performed for each treatment. The forces *F_T_* and *F_E_* were measured using a digital force gauge with an accuracy of ±0.1 N.

### 2.5. Statistical Analysis

All data were statistically analyzed using CoStat software (ver. 6.400). A three-factor randomized complete block design in a factorial arrangement was applied to assess the effects of temperature, pressure, and date variety. Analysis of variance (ANOVA) was used to evaluate the significance of main effects and interactions. When significant differences were found (*p* < 0.05), means were compared using Duncan’s multiple range test. Additionally, one-way ANOVA and two-way ANOVA applying Tukey’s HSD test were used for selected comparisons, and coefficients of variation (CV) were calculated to assess data consistency. Regression and correlation analyses were also performed to explore relationships between physical properties [30].

## 3. Results

### 3.1. Physical Characteristics of Date Fruits

The average values for various physical characteristics of the date fruits, including length (*L*), width (*w*), thickness (*T*), flesh thickness (*T_f_*), mass (*m*), volume (*V*), true density (*ρ_T_*), bulk density (*ρ_b_*), moisture content (*MC*), geometric mean diameter (*d_g_*), and sphericity (S), are shown in Table 1. In Sukkari dates, a noticeable decrease is observed across various parameters as ripening progresses from Khalal to Rutab and finally to Tamar. Specifically, the fruit length decreases by approximately 8%, width by 7.7%, and thickness by 14%. Similarly, the flesh thickness follows a comparable trend, shrinking by about 8%, while the fruit’s mass and volume contract by 21% and 18%, respectively. Moisture content shows a substantial and statistically significant decline from around 61.8% to 17.3% (*p* < 0.05), marking a reduction of 44.5%. This statistically considerable moisture decrease (*p* < 0.05) reflects the extensive water loss, contributing to the concentration of sugars within the fruit. The bulk density decreases slightly from 0.64 g·cm^−3^ to 0.61 g·cm^−3^, which suggests the compaction of the fruit’s tissue as the intercellular voids collapse. In contrast, the true density declines from 0.87 g·cm^−3^ to 0.83 g·cm^−3^, indicating that the mass loss due to water evaporation exceeds the fruit’s solids accumulation [31]. The geometric mean diameter in Sukkari fruits reduces by about 10.2%. At the same time, the sphericity experiences a marginal decrease of 0.02, showing that the fruit’s shape remains mostly consistent despite the reduction in overall size.

For Khalas dates, the initial dimensions are larger, with fruit length around 42.3 mm, width 18.1 mm, and thickness 14.4 mm in the Khalal stage. However, as the fruit ripens, there is a reduction of roughly 10–12% in these dimensions by the Tamar stage. Flesh thickness decreases by 15.5%, mass by 20%, and volume by 16%. Moisture content declines steeply from 73.4% to 24.6%, reflecting a 48.8% loss. Bulk density increases from 0.80 to 0.84 g·cm^−3^, which is consistent with the collapse of the porous spaces within the fruit.

On the other hand, true density decreases from 1.77 g·cm^−3^ to 1.68 g·cm^−3^, indicating a notable loss in mass relative to the fruit’s solid content. This more pronounced decrease in true density compared to Sukkari reflects Khalas’s inherently thicker cell walls and higher initial solids content, amplifying mass loss effects [32]. This significant decrease in true density, compared to Sukkari, can be attributed to the inherently thicker cell walls and higher initial solids content in Khalas dates, which amplify the effects of mass loss due to moisture evaporation. The geometric mean diameter and sphericity in Khalas dates decline by around 9% and between 0.00 and 0.02 units, respectively, suggesting that the shape remains unaffected, mainly despite the decrease in size.

The size reduction is less pronounced for Saqie dates, which are the largest of the three varieties. Length decreases by approximately 5%, width by 4.7%, and thickness by 4.8%. Flesh thickness shows a notable decrease of 17.2%, mass drops by 20.9%, and volume shrinks only by 1.9%, indicating greater resistance to volumetric collapse than the other varieties. Moisture content in Saqie dates decreases from 62.2% to 24.6%, a loss of 37.6%, somewhat less than the other two varieties. This may be due to the denser cuticle and mesocarp of Saqie dates, which act to slow the process of transpiration. Bulk density remains nearly constant, changing from 0.63 g·cm^−3^ to 0.62 g·cm^−3^. This suggests that while microscopic voids collapse, the overall packing density is maintained by forming rigid sugar matrices within the fruit. True density experiences a marked reduction, from 1.07 g·cm^−3^ to 0.86 g·cm^−3^, a 19.6% decline, which exceeds the reductions observed in Sukkari and Khalas. This significant decrease in true density may be attributed to unique solute-crystallization dynamics within the vacuoles of Saqie fruit.

Across all three varieties, moisture content exhibited the most significant relative change, and this change was strongly correlated with both bulk density and true density, with a coefficient of determination (r^2^) greater than 0.85 and a *p*-value of less than 0.001. Regression analysis revealed that every 10% reduction in moisture content resulted in an average increase of 0.03 g·cm^−3^ in bulk density and a decrease of 0.05 g·cm^−3^ in true density, regardless of variety. Furthermore, flesh thickness showed a positive correlation with both fruit volume (r = 0.92) and mass (r = 0.90), confirming that the integrity of the mesocarp plays a crucial role in determining the overall size of the fruit. The fruits’ geometric mean diameter and sphericity were also found to be tightly correlated (r = 0.98), suggesting that the genetics behind the fruit’s shape remain largely unaffected by water loss during ripening.

### 3.2. Stress–Strain Curve Analysis

**Figure 4:**
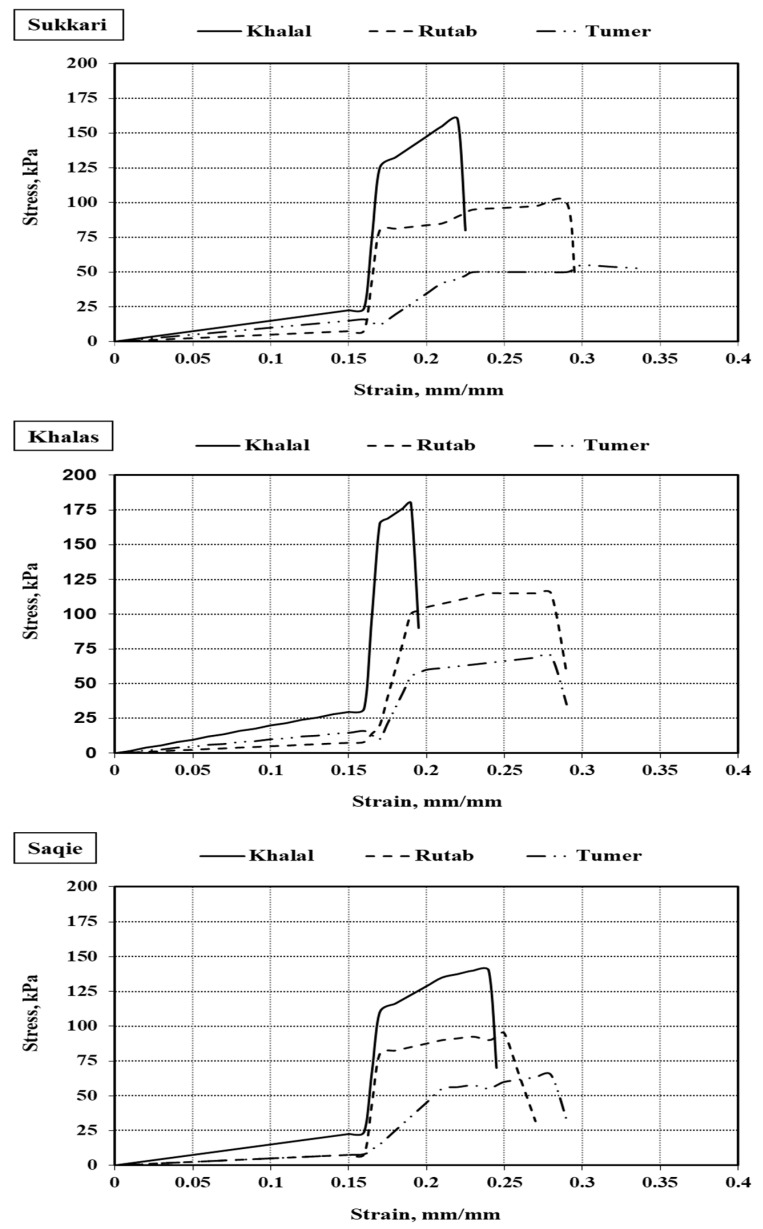
Stress–strain curves of three different date fruit varieties.

Figure 4 illustrates the stress–strain curves for the studied date varieties—Sukkari, Khalas, and Saqie—across the three primary ripening stages: Khalal, Rutab, and Tamar. The stress–strain curves reflect the mechanical behavior of the date fruits during uniaxial compression, revealing essential aspects of texture and structural evolution as ripening progresses. These curves highlight the relationship between stiffness, strength, and deformability, which are key indicators of fruit quality and the required handling protocols for postharvest processing.

In Sukkari dates, the stress–strain curve at the Khalal stage shows a steep initial slope, indicating an elastic modulus in the range of 160–180 kPa, characteristic of high tissue rigidity, which is due to the elevated moisture content of approximately 61.8% and a firm cell structure. Yield stress is observed around 120 kPa, and fracture stress ranges from 150 to 160 kPa, with a fracture strain of 0.22. This low fracture strain indicates limited plastic deformation, suggesting that the fruit resists mechanical damage at this early stage. The softening tissue becomes evident as the ripening progresses to the Rutab stage. The elastic modulus drops to 100–120 kPa, yield stress decreases to 75–85 kPa, fracture stress reduces to about 95–100 kPa, and fracture strain increases to 0.30. These changes indicate that the tissue becomes more ductile and exhibits a broader plastic region before failure. In the Tamar stage, the curve flattens significantly, with the elastic modulus falling to 55–65 kPa and fracture stress around 50–55 kPa. The fracture strain increases to approximately 0.36, indicating increased deformability and significant tissue softening, corresponding with the moisture content reduction to 17.3%. The mechanical evolution of Sukkari dates aligns with findings by Soomro et al. [33], who documented progressive softening in date tissues as they mature. Sukkari exhibits a more gradual transition than other varieties, possibly due to differences in cell wall composition.

For Khalas dates, the mechanical properties are the most rigid among the studied varieties at the Khalal stage. The elastic modulus is estimated at 200–220 kPa, significantly higher than Sukkari. Yield stress and fracture stress are recorded at 150–160 kPa and up to 180 kPa, respectively. In contrast, fracture strain remains low at approximately 0.18, indicating a stiff and brittle response with minimal plastic deformation. This behavior is consistent with Khalas’s compact tissue morphology and high true density of 1.77 g·cm^−3^. At the Rutab stage, the Khalas fruit undergoes moderate softening; the elastic modulus decreases to 130–140 kPa, fracture stress reduces to about 115 kPa, and yield stress decreases to approximately 100 kPa. Fracture strain increases to 0.28, indicating improved ductility while still maintaining considerable strength. By the Tamar stage, Khalas fruits show a significant loss in mechanical integrity, with the elastic modulus reduced to around 70 kPa and fracture stress near 65–70 kPa. The curve becomes more gradual, and fracture strain rises to about 0.35, indicating more pronounced viscoelastic behavior as cell wall cohesion diminishes. Despite the moisture content dropping to 24.6%, Khalas retains higher mechanical resistance compared to Sukkari in the final stage, demonstrating its ability to maintain structural integrity and suitability for extended shelf life and mechanical handling.

The stress–strain behavior of the Saqie dates is displayed in Figure 4. This variety demonstrates an intermediate mechanical response between Sukkari and Khalas. At the Khalal stage, the elastic modulus for Saqie is approximately 150–160 kPa, with yield stress around 110 kPa and fracture stress between 130 and 140 kPa. The fracture strain is about 0.24, which is slightly higher than Khalas, suggesting a relatively more compliant tissue while still firm and resistant to deformation. This profile reflects a relatively uniform structure with moderate moisture content (62.18%) and a true density of approximately 1.07 g·cm^−3^. At the Rutab stage, the mechanical properties gradually decline: the elastic modulus drops to 100–110 kPa, fracture stress reduces to 90–95 kPa, and yield stress decreases to about 80–85 kPa. The fracture strain increases to 0.30, which is similar to the changes observed in Sukkari at the same stage, indicating a similar degree of softening. By the Tamar stage, the elastic modulus of Saqie remains higher than that of Sukkari and slightly lower than Khalas, approximately 75 kPa, with a fracture stress estimated at 65 kPa. The fracture strain increases to 0.33. This gradual decline in mechanical properties suggests that Saqie maintains better structural integrity during ripening, which supports its variety-specific resilience to softening, as reported by Hassan et al. [18], who also noted the varying rates of softening among date varieties during maturation.

Across all three varieties, the progression from Khalal to Tamar is consistently associated with reduced elastic modulus, fracture stress, and increased fracture strain. These mechanical changes correlate strongly with the decrease in moisture content and the corresponding decline in tissue density, emphasizing the role of cellular turgidity and microstructural cohesion in maintaining mechanical performance.

To statistically validate the observed mechanical differences among varieties and ripening stages, a one-way analysis of variance (ANOVA) was performed on the extracted mechanical parameters, including elastic modulus, yield stress, fracture stress, and fracture strain. The study revealed statistically significant differences (*p* < 0.05) for all variables across the three maturity stages for each variety. Where significant, Duncan’s multiple range test was applied for pairwise comparisons. These results confirm that the changes in mechanical response during ripening are visually evident from the stress–strain curves and statistically supported.

Moreover, Pearson correlation analysis was conducted to explore the relationships between mechanical parameters and physical fruit characteristics. A strong negative correlation (r = −0.87) was found between the elastic modulus and moisture content, indicating that softer tissue at later stages corresponds with lower internal water levels. Similarly, fracture stress was positively correlated with flesh thickness and actual density (r = 0.81 and r = 0.76, respectively), highlighting the role of internal tissue structure in resisting compression. These statistically supported relationships suggest that mechanical properties such as elasticity and fracture behavior could serve as quality indicators, particularly in assessing textural integrity and postharvest handling suitability for different varieties.

The significant varietal differences observed in deformation behavior suggest that stress–strain curve parameters may contribute to a mechanical quality index that reflects resistance to handling damage and consumer-desired texture. For example, fruits maintaining higher fracture stress and moderate elasticity at the Tamar stage, like Khalas, could be considered more robust during packaging and transport. These findings can inform ripeness-based sorting protocols and support variety-specific postharvest strategies tailored to preserving quality throughout the supply chain [34].

### 3.3. The Dynamic Coefficient of Friction (μd)

**Figure 5:**
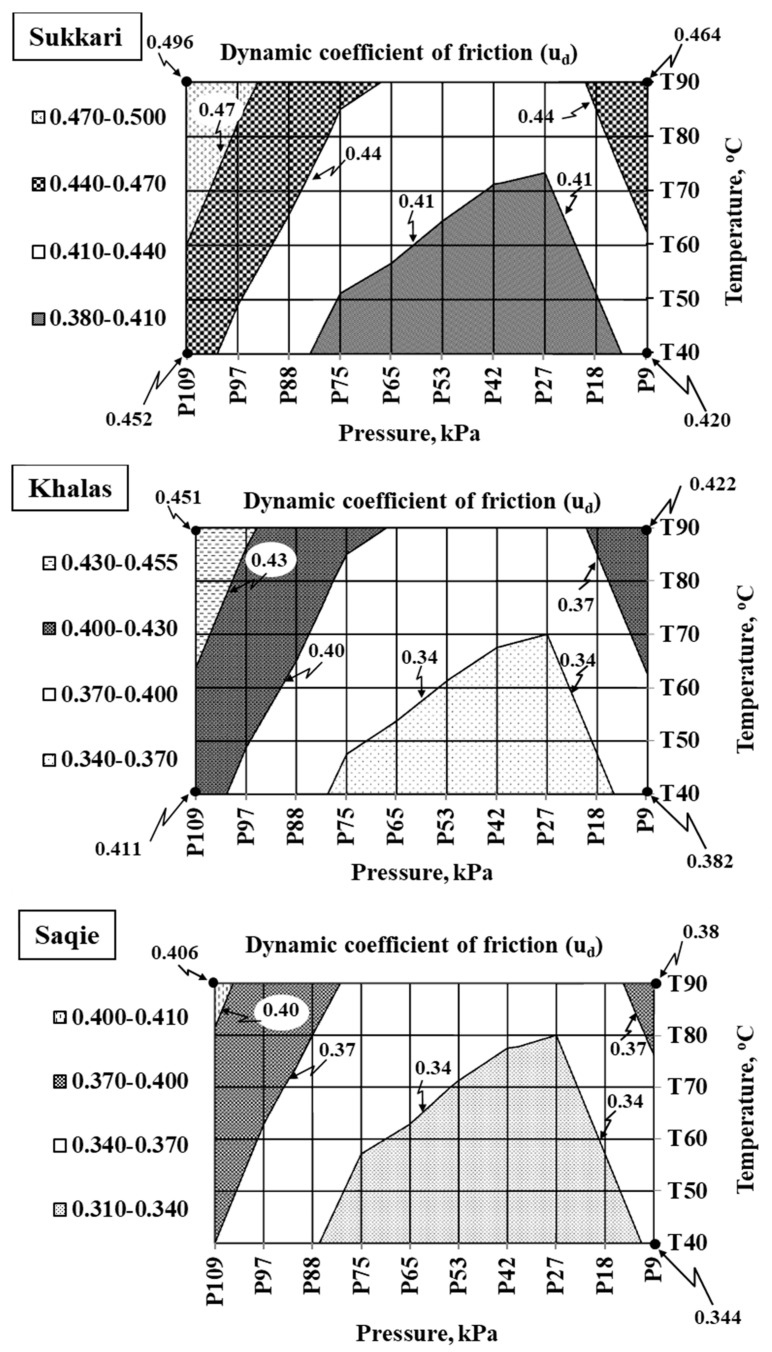
Dynamic coefficient of friction of date fruits under varying pressure and temperature at the Tamar ripening stage.

The average values of the dynamic coefficient of friction (*μd*) for date fruits across different varieties and varying conditions of temperature and pressure are illustrated in Figure 5. For the Sukkari variety at the Tamar stage, a clear increasing trend in *μd* was observed with rising temperature and applied pressure. At a lower temperature of 40 °C, the dynamic coefficient of friction ranged from 0.420 at 9 kPa to 0.452 at 109 kPa. As the temperature increased to 50 °C and above, the *μd* showed a progressive increase, reaching a maximum value of 0.496 at 90 °C under 109 kPa. This indicates that Sukkari fruits become increasingly adhesive as the temperature rises, which can be attributed to the softening of the epidermis and the migration of surface moisture. The relatively small standard deviation among the replicates further suggests that Sukkari’s mechanical behavior under thermal stress is consistent. These observations align with the findings of Soomro et al. [33], who reported that surface stickiness and frictional resistance in date fruits increase significantly with temperature due to plasticization and internal moisture redistribution. Additionally, the higher sugar content and semi-dry texture of Sukkari at the Tamar stage contribute to increased surface adhesion and friction, particularly at higher temperatures.

A similar trend was observed for the Khalas variety, though the friction increase was somewhat less pronounced. The *μd* values at 40 °C varied between 0.382 and 0.411, with the coefficient gradually increasing with higher temperatures and pressures. At 90 °C and 109 kPa, the peak *μd* reached 0.451. Compared to Sukkari, Khalas exhibited a more moderate rate of increase in friction with temperature, which could be due to the softer and more homogeneous texture of Khalas dates at full ripeness. This texture results in relatively lower resistance to motion.

Statistically, Khalas showed a slightly wider spread in the friction data at higher temperatures, suggesting that its mechanical response was more variable. This could be attributed to the breakdown of microstructures and partial liquefaction of internal tissues, a phenomenon previously documented by Al-Shahib and Marshall [35], who detailed the compositional differences of Khalas compared to other date varieties. At the Tamar stage, Khalas dates, which are characterized by a higher moisture content, likely experience a combined effect of surface smoothness and increased lubrication at intermediate pressures. However, as the temperature rises, the fruit becomes excessively soft and sticky, causing friction to increase again at the highest thermal levels.

For the Saqie variety, the dynamic coefficient of friction consistently remained the lowest among the three varieties across all temperature and pressure combinations. At 40 °C, *μd* ranged from 0.344 to 0.370 and increased moderately, reaching a maximum of 0.406 at 90 °C and 109 kPa. Unlike Sukkari and Khalas, the increase in *μd* with temperature and pressure for Saqie was more gradual, suggesting that Saqie has a more stable surface under heating. This implies that the epidermis of Saqie dates is less thermally sensitive and maintains better mechanical integrity even under elevated temperatures.

Devahastin [36] also highlighted that varieties with lower moisture contents, such as Saqie, exhibit less pronounced changes in mechanical properties during thermal processing, which supports these findings. The relatively lower friction coefficients indicate that Saqie fruits would require less energy for movement across mechanical conveyors or during other handling operations, making them potentially more advantageous in industrial processing.

Statistical analysis using analysis of variance (ANOVA) revealed significant differences (*p* < 0.05) between the three date varieties—Sukkari, Khalas, and Saqie—in terms of the dynamic coefficient of friction under the tested conditions. Sukkari consistently recorded the highest friction values, followed by Khalas, with Saqie exhibiting the lowest. The relationship between temperature, pressure, and friction appeared more linear for Sukkari and Khalas.

In contrast, Saqie showed a less steep increase in friction, indicating better thermal resistance and stability. The varying compositions of the varieties can explain these differences in frictional behavior. Sukkari and Khalas, with their higher sugar and moisture content, experience more surface softening and stickiness at elevated temperatures. At the same time, Saqie’s firmer texture and lower water activity allow it to maintain lower friction levels.

The mechanical implications of these results are crucial for the postharvest handling and processing of date fruits. Higher dynamic coefficients of friction can lead to increased mechanical resistance, necessitating greater energy input in conveying and packaging systems, and could result in more mechanical damage to the fruits. Sukkari, due to its higher *μd*, may require specialized equipment with lubricated surfaces or controlled temperature environments to minimize friction and reduce wear. On the other hand, Saqie, with its lower friction characteristics, may be processed with less complexity, making it more efficient in industrial settings.

Pearson correlation analysis was conducted between *μd* and selected quality-related variables such as moisture content, true density, and fruit firmness to further support the interpretation of these trends. A strong positive correlation was observed between μd and surface moisture (r = 0.83), indicating that water migration significantly enhances surface adhesion during thermal exposure. Conversely, μd exhibited a negative correlation with fruit firmness (r = − 0.79), especially at higher temperatures, reflecting the softening of the fruit’s outer layer and increased contact area with frictional surfaces. These statistically validated relationships reinforce the role of μd as a functional indicator of handling performance. Specifically, varieties with high μd values under elevated temperatures—such as Sukkari—may be more prone to sticking and damage on dry conveyor belts, whereas those with low μd—such as Saqie—are better suited for high-throughput mechanical systems. From an industry standpoint, the dynamic coefficient of friction can be integrated into a broader quality index that reflects internal texture and surface behavior during mechanical handling. Varieties exhibiting stable μd values across temperature and pressure ranges can be prioritized for automation-based postharvest systems, while more friction-sensitive fruits may require adjustments in equipment design. These insights are valuable for designing sorting, packaging, and transport strategies tailored to each variety’s mechanical profile [37].

## 4. Discussion

This study aimed to investigate the dynamic friction and physico-mechanical properties of date fruits for food quality enhancement, with a particular focus on three varieties, Sukkari, Khalas, and Saqie, for conserving the keeping and appearance qualities. The research encompassed three primary sections: physical properties, stress–strain behavior, and dynamic coefficient of friction of the fruits across different ripening stages. The results in this study align with previous research on the effects of ripening on the physical characteristics of date fruits. The decrease in fruit size, mass, and moisture content during ripening has been well-documented, with the loss of water being a critical determinant of both bulk and true density. Studies such as those by Al-Farsi and Lee [38] on Medjool dates have demonstrated proportional reductions in fruit dimensions and moisture content during ripening, driven by cell wall loosening and the loss of turgor. Similarly, Al-Eid et al. [39] reported that bulk density increased and true density decreased in Deglet Nour and Medjool varieties, with these changes attributed to the collapse of intercellular spaces and the concentration of sugars within the fruit’s tissue.

These findings are consistent with the observations in this study, particularly for the Khalas and Saqie varieties. The significant decline in moisture content across all three varieties is a crucial factor in the physical transformations observed. This trend corresponds with Al-Dashti et al. [40], who noted that Khalas dates lost approximately 45% of their water content between the Khalal and Tamar stages, leading to a corresponding decrease in density. The mechanisms behind this moisture loss are likely related to both the physical characteristics of the fruit’s skin and the cell wall structure, with varieties like Saqie showing resistance to volumetric collapse due to their denser cuticle and mesocarp, which help slow the moisture evaporation process [41]. Despite these reductions in size and moisture, the dates’ geometric mean diameter and sphericity exhibited minimal changes during ripening, suggesting that the fruit shape remains largely preserved even as the fruit shrinks. These findings are consistent with previous studies showing fruit shape stability across different ripening stages and varieties [17,31]. The dynamics of true density during ripening appeared to vary across varieties. While a marked reduction in true density was observed for Saqie dates, other studies, such as Hazbavi et al. [42], reported a slight increase in proper density in certain varieties like Deglet Nour at the Tamar stage. This discrepancy could be attributed to differences in sugar crystallization kinetics and cell wall composition among varieties, influencing the deposition of solids in the fruit’s vacuoles. The variety × maturity interactions identified in this study are essential for optimizing postharvest handling. The moisture content and density interactions suggest that drying protocols optimized for one variety, such as Sukkari, may not be suitable for others, like Khalas or Saqie, which could lead to over-drying or under-drying, potentially affecting texture and shelf life [10].

The stress–strain behavior of Sukkari, Khalas, and Saqie varieties revealed distinct mechanical properties that change significantly as the fruit ripens. These changes, driven primarily by moisture loss and the weakening of cell wall integrity, result in the progressive softening of the fruit tissue [43]. In Sukkari, a substantial decrease in mechanical resistance from Khalal to Tamar stage was observed, with the elastic modulus dropping from 160–180 kPa to 55–65 kPa and fracture stress reducing from 150–160 kPa to 50–55 kPa. These mechanical changes correspond to a decrease in moisture content from 61.8% at Khalal to 17.3% at Tamar. The increase in fracture strain from 0.22 to 0.36 highlights the increased deformability of the tissue as it softens. These findings align with Soomro et al. [33], who reported similar mechanical trends during maturation. Sukkari undergoes a more gradual textural transition than other varieties, likely due to its cell wall composition and structure. At the Khalal stage, Sukkari fruits exhibit resistance to mechanical damage, as indicated by the relatively low fracture strain. Khalas, on the other hand, maintained a high degree of rigidity at the Khalal stage, with an elastic modulus of 200–220 kPa and a fracture stress of 150–160 kPa. This high mechanical resistance reflects the dense internal structure and high true density of Khalas, characteristic of this variety. As ripening progresses to the Tamar stage, Khalas shows a more gradual decrease in mechanical resistance, with the elastic modulus dropping to about 70 kPa and fracture stress reducing to 65–70 kPa. Despite the significant reduction in moisture content to 24.6%, Khalas retains relatively high mechanical resistance compared to Sukkari, suggesting stronger structural retention during ripening, consistent with Wilkins [44]. Saqie exhibited intermediate mechanical properties, with an elastic modulus at the Khalal stage of approximately 150–160 kPa, comparable to Sukkari but lower than Khalas. The yield stress and fracture stress at the Khalal stage (110 kPa and 130–140 kPa, respectively) indicate firm tissue, while the fracture strain of 0.24 suggests slightly more deformability compared to Khalas. The mechanical properties of Saqie decline gradually, maintaining better structural integrity compared to Sukkari, which experiences a more abrupt drop in mechanical properties. This aligns with findings by Hassan et al. [18], who noted varying softening rates in different date varieties during ripening, with Saqie demonstrating enhanced resilience to softening. The general trend across all three varieties shows a consistent reduction in elastic modulus and fracture stress, coupled with increased fracture strain, as moisture content decreases and cell wall integrity is compromised. These mechanical changes are linked to structural modifications as the fruit ripens. According to Mohsenin [13], tissue softening in fruits is primarily driven by the breakdown of cell walls and intercellular adhesion, which is reflected in the observed mechanical behavior of the date fruits. The transition from a brittle to a more ductile response in the stress–strain curves suggests that different handling strategies are required at each ripening stage. Khalal fruits, being firmer, are better suited for mechanical processing, while the softer Rutab and Tamar stages necessitate more delicate handling to prevent tissue damage. The mechanical properties of the date fruits can guide the design of optimized harvesting, sorting, packaging, and processing systems, as they directly influence the quality of the fruit during postharvest operations [4].

The dynamic friction coefficient of date fruits increased with temperature and applied pressure, showing significant varietal differences. Sukkari exhibited the highest *μd* values, followed by Khalas, with Saqie displaying the lowest friction values. These findings align with Soomro et al. [33] and Al-Shahib and Marshall [35], who noted that temperature and moisture content influence the frictional properties of date fruits. For Sukkari, the friction increased as the fruit softened with ripening, likely due to higher sugar content and moisture redistribution, contributing to a more adhesive surface [45]. Khalas exhibited a lower rate of increase in friction, possibly due to its more homogeneous texture, resulting in a more gradual softening. Saqie displayed lower friction with a firmer texture and lower moisture content, which could be advantageous in postharvest processing as it requires less energy for handling. These differences in frictional characteristics suggest that mechanical handling equipment for Sukkari may need to be designed with lubricated surfaces or temperature-controlled environments to minimize friction and wear [46]. In contrast, Saqie, with its lower friction, may be easier to handle mechanically, requiring less complex systems [47].

Understanding the mechanical properties of different date varieties is essential for the design of efficient harvesting, sorting, packaging, and processing systems. So, this study highlights the complex relationship between date fruits’ physical, mechanical, and frictional properties during ripening [48]. Interestingly, higher friction values indicate greater resistance to sliding motion, which may influence conveyor belt design, sorting efficiency, and energy requirements during transportation [49]. This property affects processing steps like milling or compression, where varieties with higher stress–strain tolerance may require adjusted pressure settings to avoid damage. Varieties with lower stress–strain tolerance may need gentler handling to prevent mechanical damage [50]. These differences impact packaging density, susceptibility to bruising, and compatibility with automated grading systems [51]. These results emphasize the need for tailored postharvest strategies to preserve quality, reduce waste, and improve operational efficiency across different agricultural varieties. Closely, the significant variety-specific differences observed in the dynamic coefficient of friction, stress–strain behavior, and physical characteristics underscore the importance of considering these factors in postharvest handling and processing.

## 5. Conclusions

This study has successfully evaluated the physico-mechanical properties of three date varieties—Sukkari, Khalas, and Saqie—during their ripening stages, aiming to enhance food quality through a deeper understanding of the mechanical attributes of date fruits. The findings reveal significant variety-specific differences, highlighting each variety’s unique structural and mechanical responses to ripening processes. Significant differences (*p* < 0.05) among varieties in key stress–strain parameters, reflecting their distinct structural reactions. The Sukkari variety demonstrated the greatest softening, with a marked reduction in stress–strain parameters due to moisture loss and weakening of cell walls. In contrast, Khalas showed a more gradual decline in stress–strain values, reflecting its higher structural resilience during ripening. Saqie exhibited the most stable mechanical properties, with minimal changes in stress–strain behavior and moisture content, due to its firmer, denser texture. The study also presented novel insights into the dynamic coefficient of friction, which increased with temperature and pressure across all varieties. Sukkari exhibited the highest friction values, followed by Khalas, and Saqie showed the lowest. This frictional trend aligns with various mechanical behaviors, which have practical implications for their processing and handling in the food industry. The novelty of this research lies in its comprehensive evaluation of both stress–strain behavior and friction properties, which are crucial for optimizing the mechanical handling and processing of date fruits to minimize damage and improve efficiency. Future studies should explore the microstructural changes within date fruits at the cellular level and their relationship with mechanical properties, especially stress–strain behavior, to further refine postharvest processing techniques. Additionally, research into variety-specific processing methods could enhance the quality and marketability of date-based products, offering valuable insights into food quality optimization.

## Figures and Tables

**Table 1:** Physical characteristics of three different date palm fruits at different ripening stages, (mean ± SD), *n* = 20.

Property	Sukkari			Khalas			Saqie		
	Khalal	Rutab	Tamar	Khalal	Rutab	Tamar	Khalal	Rutab	Tamar
*L*, mm	39.25 ± 1.93 ^a^	36.40 ± 1.77 ^b^	36.03 ± 1.75 ^b^	42.26 ± 1.95 ^a^	38.19 ± 177.9 ^b^	38.04 ± 1.75 ^b^	51.60 ± 0.75 ^a^	50.13 ± 0.73 ^b^	49.15 ± 0.72 ^c^
*w*, mm	31.75 ± 1.45 ^a^	29.49 ± 1.34 ^b^	29.31 ± 1.32 ^b^	18.06 ± 0.85 ^a^	16.31 ± 0.77 ^b^	16.21 ± 0.76 ^b^	26.55 ± 0.80 ^a^	25.79 ± 0.77 ^b^	25.29 ± 0.76 ^b^
*T*, mm	32.01 ± 1.42 ^a^	28.58 ± 1.27 ^b^	27.43 ± 1.22 ^c^	14.38 ± 0.66 ^a^	13.30 ± 1.46 ^b^	13.29 ± 1.28 ^b^	23.37 ± 1.65 ^a^	22.48 ± 1.59 ^b^	22.26 ± 1.75 ^b^
*T_f_*, mm	6.08 ± 0.30 ^a^	5.67 ± 0.28 ^b^	5.57 ± 0.28 ^b^	2.20 ± 0.16 ^a^	2.14 ± 0.05 ^a^	1.86 ± 0.02 ^b^	8.09 ± 0.39 ^a^	6.87 ± 0.33 ^b^	6.70 ± 0.47 ^b^
*m*, g	16.14 ± 2.39 ^a^	13.88 ± 1.34 ^b^	12.67 ± 0.29 ^c^	10.01 ± 0.45 ^a^	8.50 ± 0.38 ^b^	8.00 ± 0.36 ^c^	16.73 ± 1.12 ^a^	14.22 ± 0.96 ^b^	13.22 ± 0.87 ^c^
*V*, cm^3^	18.82 ± 2.31 ^a^	16.56 ± 1.78 ^b^	15.39 ± 1.87 ^b^	5.78 ± 0.79 ^a^	5.20 ± 0.71 ^b^	4.86 ± 0.66 ^b^	15.70 ± 1.33 ^a^	15.69 ± 1.34 ^a^	15.40 ± 1.32 ^a^
*ρ_T_*, g·cm^−3^	0.87 ± 0.17 ^a^	0.85 ± 0.16 ^a^	0.83 ± 0.10 ^a^	1.77 ± 0.27 ^a^	1.67 ± 0.26 ^a^	1.68 ± 0.26 ^a^	1.07 ± 0.11 ^a^	0.91 ± 0.09 ^b^	0.86 ± 0.09 ^b^
*ρ_b_*, g·cm^−3^	0. 64 ± 0.02 ^a^	0. 62 ± 0.01 ^b^	0. 61 ± 0.03 ^b^	0. 80 ± 0.08 ^a^	0. 83 ± 0.09 ^a^	0. 84 ± 0.09 ^a^	0.63 ± 0.02 ^a^	0.62 ± 0.01 ^a^	0.62 ± 0.03 ^a^
*MC*, %	61.80 ± 4.37 ^a^	37.08 ± 2.62 ^a^	17.30 ± 1.22 ^c^	73.37 ± 1.96 ^a^	51.87 ± 2.1 ^b^	24.63 ± 3.89 ^c^	62.18 ± 3.68 ^a^	25.33 ± 1.49 ^b^	24.62 ± 1.49 ^b^
*d_g_*, mm	34.16 ± 1.40 ^a^	31.30 ± 1.35 ^b^	30.71 ± 1.27 ^b^	22.22 ± 1.03 ^a^	20.21 ± 1.08 ^a^	20.14 ± 0.87 ^b^	31.73 ± 0.83 ^a^	30.73 ± 0.81 ^b^	30.22 ± 0.79 ^b^
*S*,	0.87 ± 0.02 ^a^	0.86 ± 0.02 ^b^	0.85 ± 0.02 ^b^	0.53 ± 0.00 ^a^	0.53 ± 0.02 ^a^	0.53 ± 0.02 ^a^	0.62 ± 0.02 ^a^	0.61 ± 0.02 ^a^	0.62 ± 0.02 ^a^

*L* = Fruit length, *w* = Fruit width, *T* = Fruit thickness, *T_f_* = Flesh thickness, *m* = Fruit mass, *V* = Fruit volume, *ρ_T_* = True density, *ρ_b_* = Bulk density, *MC* = Moisture content, *d_g_* = Geometric mean diameter, *S* = Degree of sphericity, ^a,b,c^: There is no significant difference (*p* > 0.05) between any two means for each species, within the same row have the same superscript letter within the same date variety.

## Data Availability

The original contributions presented in this study are included in the article/Supplementary Materials. Further inquiries can be directed to the corresponding author.

## Supplementary Materials

The following supporting information can be downloaded at https://www.mdpi.com/article/10.3390/foods14111838/s1, Figure S1: Shows the main ripening stages of three varieties of date palm (Sukkari, Khalas and Sakai).
