# Supplementary material for: Assessing the Physico-Mechanical Properties of Three Date Fruit Varieties for Conserving the Keeping and Appearance Qualities
Source: Foods. 2025 May 22;14(11):1838. doi: 10.3390/foods14111838 (PMC12155257; doi:10.3390/foods14111838)
Supplement: Supplementary file 1 [file foods-14-01838-s001.zip › foods-3644751-supplementary.pdf]

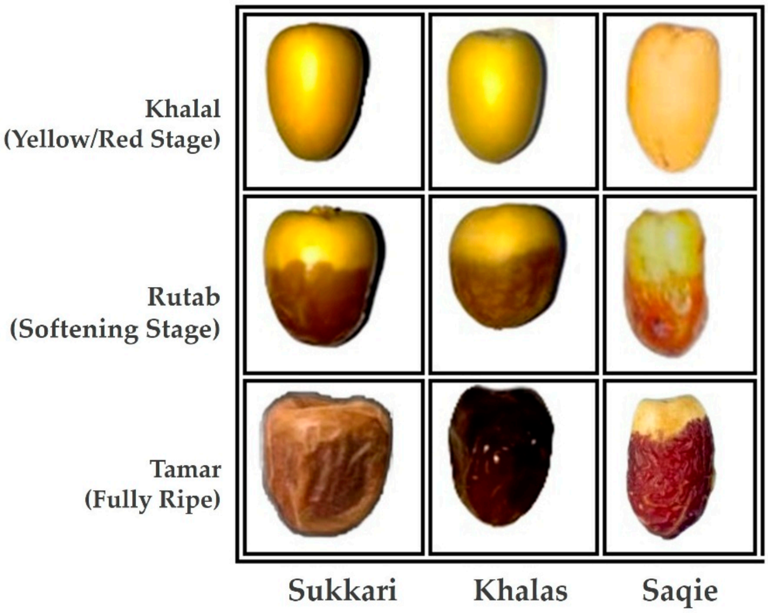

Figure S1. Shows the main ripening stages of three varieties of dates palm (Sukkari, Khalas and Sakai).
